# IDP-LM: Prediction of protein intrinsic disorder and disorder functions based on language models

**DOI:** 10.1371/journal.pcbi.1011657

**Published:** 2023-11-22

**Authors:** Yihe Pang, Bin Liu

**Affiliations:** 1 School of Computer Science and Technology, Beijing Institute of Technology, Beijing, China; 2 Advanced Research Institute of Multidisciplinary Science, Beijing Institute of Technology, Beijing, China; Tel Aviv University, ISRAEL

## Abstract

Intrinsically disordered proteins (IDPs) and regions (IDRs) are a class of functionally important proteins and regions that lack stable three-dimensional structures under the native physiologic conditions. They participate in critical biological processes and thus are associated with the pathogenesis of many severe human diseases. Identifying the IDPs/IDRs and their functions will be helpful for a comprehensive understanding of protein structures and functions, and inform studies of rational drug design. Over the past decades, the exponential growth in the number of proteins with sequence information has deepened the gap between uncharacterized and annotated disordered sequences. Protein language models have recently demonstrated their powerful abilities to capture complex structural and functional information from the enormous quantity of unlabelled protein sequences, providing opportunities to apply protein language models to uncover the intrinsic disorders and their biological properties from the amino acid sequences. In this study, we proposed a computational predictor called IDP-LM for predicting intrinsic disorder and disorder functions by leveraging the pre-trained protein language models. IDP-LM takes the embeddings extracted from three pre-trained protein language models as the exclusive inputs, including ProtBERT, ProtT5 and a disorder specific language model (IDP-BERT). The ablation analysis shown that the IDP-BERT provided fine-grained feature representations of disorder, and the combination of three language models is the key to the performance improvement of IDP-LM. The evaluation results on independent test datasets demonstrated that the IDP-LM provided high-quality prediction results for intrinsic disorder and four common disordered functions.

## Author summary

The intrinsically disordered proteins (IDPs) and regions (IDRs) are functionally important proteins and regions without stable three-dimensional structures under the native physiologic conditions. They are widespread in proteome and performed many critical biological functions in organisms. The structural and functional abnormalities of IDRs/IDPs typically cause many severe diseases in humans. Therefore, computational identification of the intrinsic disorder and its functions in protein is important for a comprehensive understanding of the protein structure-function mechanism, thereby facilitating the research on disease and drug discovery. Recently, the pre-trained protein language model has been shown to be effective in discovering the structure and function information from the massive amino acid sequences. This allows us to uncover the biological properties of intrinsic disorder from the sequences through the language models (LMs). In this study, we proposed a disorder and its functions predictor referred to as IDP-LM by applying the pre-trained protein LMs. The IDP-LM takes the embeddings extracted from three pre-trained protein language models as the exclusive inputs, including ProtBERT and ProtT5 and a disordered specific language model (IDP-BERT). The IDP-BERT provides fine-grained feature representations for disorder at both the residue and sequence level, and its combination with two ProtTrans LMs is the key to the performance improvement of IDP-LM. We evaluated the performance of IDP-LM for disorder and disorder function prediction on Critical Assessment of protein Intrinsic Disorder (CAID) dataset and TE176 independent test dataset, and the results demonstrated that IDP-LM provided high-quality prediction results for both intrinsic disorder and four common disordered functions, and significantly outperformed other comparative predictors.

## Introduction

The protein segments that lack stable three-dimensional structures under the native physiologic conditions are referred to as intrinsically disordered regions (IDRs). Intrinsically disordered proteins (IDPs) with IDRs are widespread in nature, for instance, a larger fraction of proteins in eukaryotic organisms are disordered [1,2]. Although IDP/IDRs lack of well-defined structural conformation, they performed many critical biological functions, such as being post-translational modification sites [3], regulation of signaling pathways [4], and mediating the phase separation process [5,6]. The functional importance of IDP/IDRs makes them associated with the pathogenesis of many severe diseases in humans [7–9]. Exploring the intrinsic disorder and its functions in protein leads to a deeper understanding of the protein structure-function mechanism, thereby facilitating the research on disease and drug discovery [10,11].

The classical protein structure-function paradigm indicated that all information about protein structure and function is encoded in their primary amino acid sequences. Over the past decades, the exponential growth in the number of proteins with known amino acid sequences deepens the gap between unannotated and experimentally characterized disordered sequences [3]. Seeking the vast wealth from the enormous quantity of unlabeled sequences is critical for bridging these gaps [12]. Recently, language models (LMs) have become increasingly impactful in natural language processing (NLP) research. The LMs are able to capture the complex syntax and semantics from the large-scaled unlabeled text corpus, and have been shown to reach state-of-the-art (SOA) performance across a range of NLP tasks in practice. The protein sequences can be seen as the language of genetics sharing strong similarities with natural language [13]. The amino acid sequences adopt structures to determine specific functions, which map with the words that follow the syntax to express meanings. Their analogies have stimulated the applying LMs to discover the structure and function information present in the amino acid sequences. For example, the ProtTrans [12] provided novel protein language models based on a series of Transformer architectures, and has appeared competitive in structure and function related prediction tasks, including secondary structure prediction, protein localization and membrane protein classification. The IDP/IDRs perform critical functions in organisms despite lacking well-defined structures, which has redefined the protein structure-function paradigm. The potential of LMs allows us to uncover the biological properties of intrinsic disorder from the amino acid sequences.

**Fig 1:**
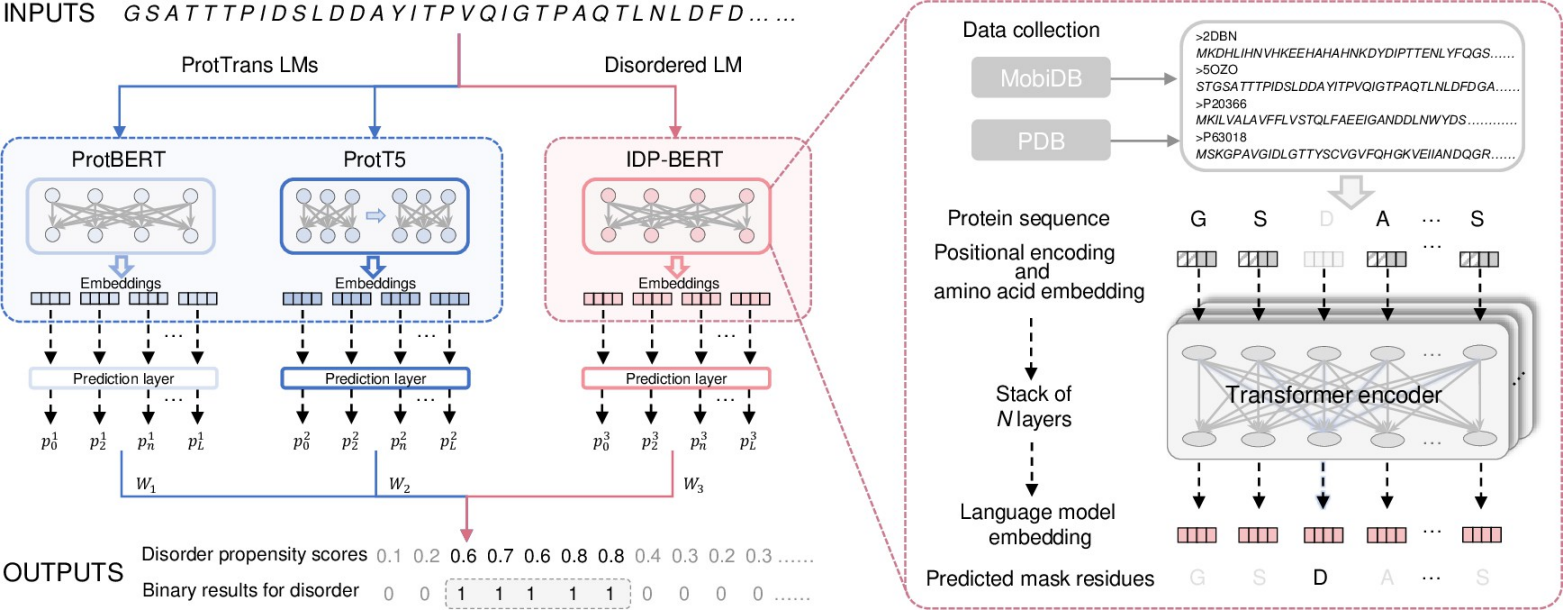
Overview of IDP-LM predictor. The input sequences were processed by three language models to generate the embedding vector for each residue. The IDP-BERT disordered language model adopts the BERT architecture by stacking multiple Transformer encoders, and it was self-supervised pre-trained with the sequences collected from the MobiDB and PDB database. Three prediction layers in IDP-LM were used to calculate per-residue propensity scores based on embeddings extracted from three language models, respectively. Then the model outputs the final propensity scores and binary results by fusing the calculations from three prediction layers.

Here, we proposed IDP-LM, a predictor by applying the pre-trained protein LMs for predicting intrinsic disorder and disorder functions of proteins (See **Fig 1**). Two pre-trained language models (ProtBERT and ProtT5) from ProtTrans that have shown especially success for protein structure and function predictions were included in the IDP-LM [12,14]. Besides, considering that the properties of disorder are particular and different from other structured regions, for example, the disorder tends to occur at the N’ and C’ terminal of sequences with a specific amino acid composition tendency [15,16], we pre-trained a disorder specific protein language model referred to as IDP-BERT, which is to capture fine-grained features of this special class of proteins/regions. The IDP-BERT employed the architecture of Bidirectional Encoder Representation (BERT) based on Transformer [17], and it was self-supervised pre-trained as masked language modelling to learn the linguistic patterns of disordered regions. The IDP-BERT provided more disorder related information at both the residue and sequence levels, and its combination with two ProtTrans LMs is able to improve the performances of IDP-LM for predicting disorder and disorder functions. We evaluated the performances of IDP-LM for disorder prediction on the Critical Assessment of protein Intrinsic Disorder (CAID) test dataset, and the results demonstrated that IDP-LM achieved competitive performance among all comparable methods in the CAID for predicting protein disordered regions, fully disordered proteins, and disordered binding subregions. Besides, we linked disorder to its functions by transferring the trained IDP-LM disorder predictor to four common disordered functions prediction including disorder protein binding, DNA binding, RNA binding, and disorder flexible linkers. Benefiting from transfer learning, the IDP-LM predictors for disorder function prediction significantly outperformed other comparative predictors in all four common disordered functions. The stand-alone package of IDP-LM is available at http://bliulab.net/IDP_LM/.

## Materials and Methods

### Datasets

#### Dataset for disordered protein language model

The protein language models (LMs) are built on the ideal that the sequence-structure-function information in proteins can be captured by effectively leveraging the massive unlabelled protein sequences. Although the functionally annotated disordered protein sequences are limited, the number of available protein sequences with detected disordered regions is sufficient. This allows us to uncover the biological properties of intrinsic disorder from the sequences through the language model. We pre-trained the disordered protein language model IDP-BERT on the sequences from the MobiDB database [18], which is a large integrated resource of disordered proteins. For obtaining high quality of protein sequences containing disordered regions, we collected all the curated and derived annotated sequences in the MobiDB but excluded their annotation information, leading to 68,700 disordered protein sequence data. Because the difference between structured and disordered is one of the most critical features for many computational prediction tasks related to intrinsic disorder [16,19–22], the IDP-BERT was pre-trained with the fully structured protein sequences to capture more differences between protein disordered and ordered. We searched all high-resolution (<2Å) protein monomers from the PDB data bank [23,24] and obtained 36,148 fully structured sequences, where all amino acid structures of these sequences have been resolved and with minimal likelihood to be disordered [19]. The combination of 68,700 disordered sequences and 36,148 structured sequences results in a total of 104,848 protein sequences for the self-supervised pre-training of disordered protein language model IDP-BERT, which are available at http://bliulab.net/IDP_LM/download/.

#### Benchmark datasets for intrinsic disorder and disorder function prediction

The information learned by pre-trained protein language models is referred as proteins’ language model embeddings [12,25], which are used as inputs for the supervised training of IDP-LM. In this study, we used two independent test datasets (CAID and TE176) to evaluated the performances of IDP-LM on intrinsic disorder and different disordered functions. Referring to the Critical Assessment of protein Intrinsic Disorder (CAID) prediction [26], the CAID dataset was used to evaluated the predictive performances on protein disordered regions, fully disordered proteins and disordered binding regions. In addition, following the study [27], the TE176 independent test set of 176 functional disordered sequences was used to perform the evaluation on four specific disorder functions, including disorder protein binding, disorder DNA/RNA bindings, and disorder flexible linkers. Besides, the training and validation dataset used for the supervised training of the proposed IDP-LMs predictor were collected from the DisProt database [28] by *Hu et al* [27]. To avoid the overestimation of predictive performance and the potential overfitting in the supervised training caused by the sequence similarity, we used the PSI-BLAST [29] searching algorithm to remove the sequences in the training and validation dataset that share sequence similarity higher than 25% to those in two independent test sets. Consequently, the resulting training set of 412 sequences and validation set of 90 sequences were used for the model parameters optimization and hyper-parameters selection, respectively. Additional descriptions of the disorder function datasets are provided in **S1 Table**. All the datasets used in this study are available at http://bliulab.net/IDP_LM/download/.

### Restrictive masked language model pre-training of IDP-BERT

The recent studies have reported that the protein language model employed the BERT architecture achieves better performance on protein function predictions than models using other architectures using the same number of pre-training sequences [12,14]. Inspired by these results, we employed the BERT framework to train the disordered protein language model, IDP-BERT. The BERT architecture refers to the Bidirectional Encoder Representation from Transformers, which is naturally suitable for masked language modelling training [30]. Unlike the BERT trained with natural language corpus and massive proteome [12], the IDP-BERT was trained, we named as a Restrictive Masked Language Model (ReMLM) for disordered regions. More specifically, the BERT model is trained to reconstruct the masked residues that are mostly located in the head and tail subsegments of sequences given the surroundings. The fundamental idea behind this novel training scheme is the observation that intrinsic disorder tends to occur at the N’ and C’ terminals of sequences and with a specific amino acid composition tendency [15,31–33].

The BERT architecture employed in IDP-BERT is almost identical to the original in NLP [17]. In IDP-BERT (see **Fig 1**), the residues were processed as the basic input units. Given a residue R_*i*_, the combination of positional encoding PE_*i*_ and amino acid embedding AE_*i*_ is used as the initial representation X_*i*_ = [PE_*i*_; AE_*i*_]. Then the inputs go through the *N* layers of Tranformer encoder blocks [30], and the hidden vector from the last layer was extracted as the language model embedding Y_*i*_ for each residue. All the model parameters were jointly optimized by minimizing the negative log likelihood of predicted masked amino acid *A*_*i*_ given the contextual $A_{\bar{M}}$ [25,34]:

$$L=-\frac{1}{N}\sum_{i=1}^{N}-log\;P(A_{i}|A_{\bar{M}})$$ (1)

where *N* denotes the maximum number of model masked residues in the input sequence. The model was implemented by PyTorch framework and trained on a single NVIDIA GeForce RTX 3080 GPU with a memory of 10GB. And the hyper-parameters of IDP-BERT are given in **S2 Table**.

### IDP-LM for disorder prediction

As the overview of IDP-LM shown in **Fig 1**, the amino acid sequence is first transformed into protein embedding vectors by three pre-trained protein language models. Two language models (ProtBERT and ProtT5) from ProtTrans [12] were used in IDP-LM, where the ProtBERT employs the BERT model was trained on UniRef100 of 216 million proteins, and the ProtT5 employs the Transformer encoder-decoder model trained with UniRef50 of 45 million proteins [12]. ProtTrans language model trained with massive scale dataset captured the comprehensive properties from the proteome. The IDP-BERT captures a fine-grained characteristic of disorder. Then three prediction layers in IDP-LM produce per-residue disorder predictions using the embeddings extracted from three language models. The prediction layers employed the bidirectional LSTM (Bi-LSTM) networks that encode the global information from the forward and backward direction of the input sequence. The preferred Bi-LSTM is inspired by previous related researches [35–37]. Given a residue R_*i*_, the disorder propensity scores calculated by three Bi-LSTM layers are represented as $p_{i}^{1},\;p_{i}^{2}$ and $p_{i}^{3}$, respectively (see **Fig 1**). Next, these propensity scores were weighted and fused into the final predictive results **P** of IDP-LM, and the optimal weights were selected by the genetic algorithm (GA) [38,39] according to the highest AUC score on the validation dataset. All the parameters of prediction layers in IDP-LM were jointly optimized by minimizing the binary cross-entropy loss [40] on the disordered validation dataset. The model hyper-parameters of IDP-LM are given in **S3 Table**.

### Transferring IDP-LM for disorder function prediction

**Fig 2:**
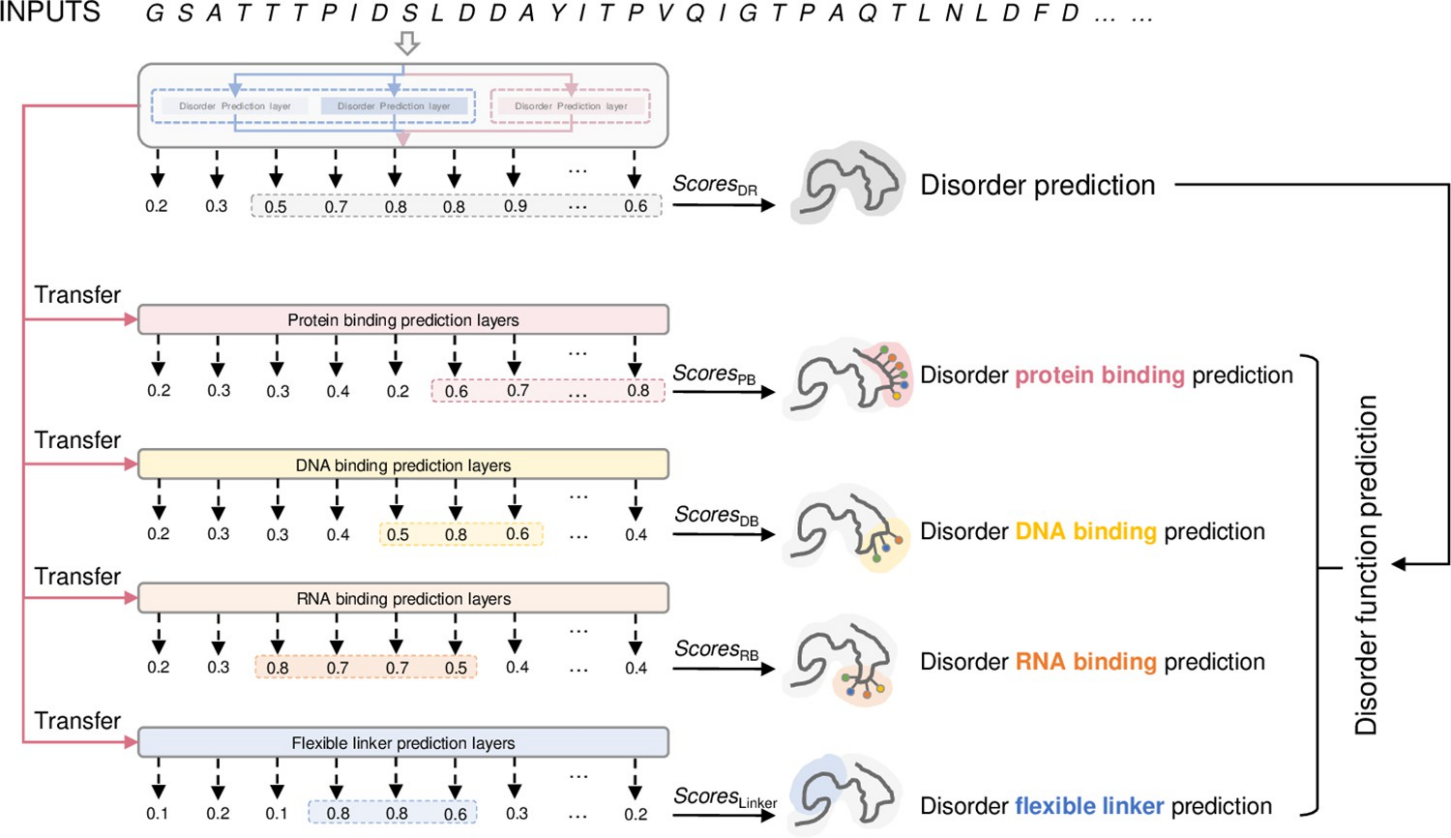
IDP-LM for disorder function prediction. The IDP-LM disorder predictor was transferred for four common disorder function predictions. Disorder prediction layers in IDP-LM were fine-tuned with disorder functions to generate *Scores*_PB_, *Scores*_DB_, *Scores*_RB_ and *Scores*_Linker_ propensity scores for predicting disorder protein binding function, disorder DNA binding function, disorder RNA binding function, and disorder flexible linker function, respectively.

Functional properties of proteins are often maintained in the natural protein amino acids sequences [25]. The language models pre-trained with massive protein sequence database discovering functional features from sequences, hence, these captured features can be used for the prediction of disordered functions. A key challenge of disordered function prediction is that the available number of disordered sequences with functional annotations is relatively small for training a computational predictor [41]. According to previous studies [22,41,42], the predictors trained with disordered sequence can be used to improve the performance of disorder function prediction via transfer learning. Therefore, in this study, we linked disorder to its function by transferring the trained IDP-LM disorder predictor to the prediction of disorder function. Specifically, the prediction layers in IDP-LM disorder predictor were separately fine-tuned with four disordered function annotated sequences, leading to four corresponding functional prediction layers for disorder protein binding, disorder DNA binding, disorder RNA binding, and disorder flexible linker (see **Fig 2**). The parameters of four functional prediction layers of IDP-LM were independently optimized using the same loss function and optimizer but different learning rates as in the disorder prediction. For the hyper-parameters of IDP-LM for disorder function prediction please refer to **S4 Table**.

### Evaluation criteria

There are two forms of results produced by the IDP-LM predictor: the real-valued propensity scores and binary classification results for disorder and disordered functions. We evaluated the real-valued prediction results with the area under the receiver operating characteristic (ROC) curve (AUC) and the maximum harmonic mean between precision and recall rate across all thresholds (F_*max*_), which fully consisted with the CAID evaluation [26]. The binary classification results were transformed from the propensity scores. The Matthews correlation coefficient (MCC) and balanced accuracy (BAC) were used to measure the binary classification results [27]:

$$MCC=\frac{TP\times TN-FP\times FN}{\sqrt{\left(TP+FP)\times (TP+FN)\times (TN+FP)\times (TN+FN\right)}}$$ (2)

$$BAC=\frac{1}{2}(\frac{TN}{TN+FP}+\frac{TP}{TP+FN})$$ (3)

where TP represents the true positives, TN represents the true negatives, FP represents the false positives, FN represents the false negatives.

## Results and discussion

### Protein language models encode the disordered properties

**Fig 3:**
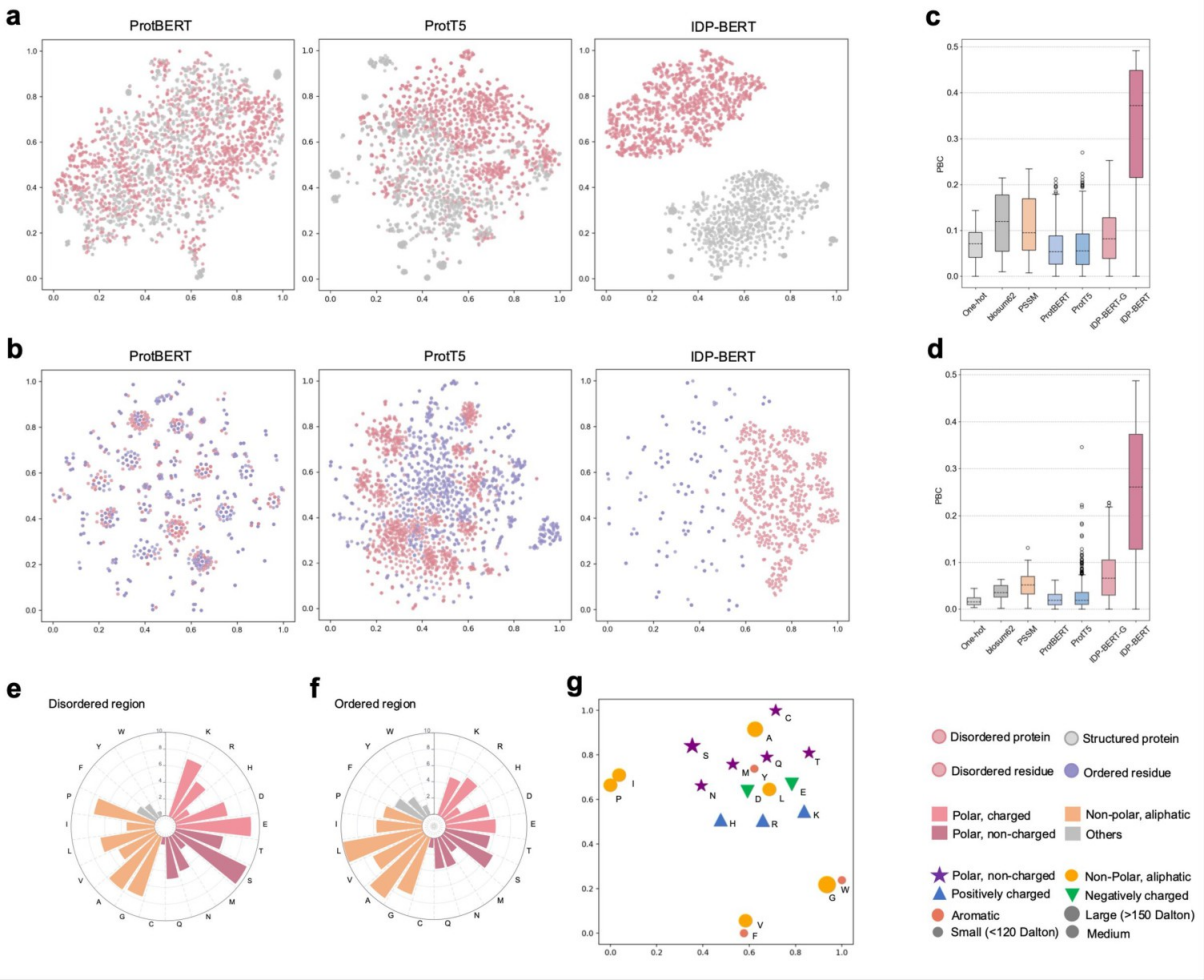
Protein disordered properties captured by language models. t-SNE projection visualization of disordered/structured proteins’ (**a**) and residues’ (**b**) embedding vectors extracted by three pre-trained protein language models, where the language model trained for disordered proteins (IDP-BERT) learned more fine-grained distinctions of disorder and order. The comparation of point-biserial correlation (PBC) scores calculated based on different feature representations of disordered proteins (**c**) and residues (**d**). We included template-free features (One-hot and blosum62), multiple sequence alignment based feature (PSSM), and pre-trained language model encodings (ProtBERT, ProtT5, IDP-BERT-G and IDP-BERT), where the IDP-BERT-G represents the features extracted from the IDP-BERT pretrained with the general mask language modelling. Higher PBC value reflects the information provided by features more relevant with disorder. According to our statistics in the DisProt database, disordered regions (**e**) are rich in polar residues compared with the ordered regions (**f**). The t-SNE projections of amino acids encoding vectors captured by IDP-BERT in 2D space conform with their biochemical properties (**g**).

To investigate how the pre-trained protein language models learn the properties of protein disorder, we visualized the embedding vectors captured by three language models in 2D space by using the t-SNE projection [43]. The output hidden vectors from the last layer of pre-trained language model are extracted as the residue-level embeddings, and the average pooling of all residue embeddings is used as the sequence-level embeddings. We randomly selected 500 fully ordered sequences and 500 disordered sequences from the pre-trained dataset. The embedding projection results derived by the three language models for a total of 1000 sequences were shown in **Fig 3A**. Subsequently, we randomly selected 500 disordered residues and 500 ordered residues in the above sampled disordered sequences, and the embedding projection of 1000 residues extracted from the three language models were shown in **Fig 3B**. Compared to the other two language models, embeddings derived from IDP-BERT are more clustered within ordered and disordered sequences/residues, and more discriminative between ordered and disordered sequences/residues. Furthermore, we utilized the point-biserial correlation (PBC) scores [20,21,44] to quantify the correlations between different feature representations and these sampled disordered residues and sequences:

$$PBC=\frac{m^{1}-m^{0}}{s_{n}}\sqrt{\frac{n^{0}\times n^{1}}{n\times n}}$$ (4)

where *m*^0^ and *m*^1^ indicate the mean values of embedding vectors for ordered and disordered proteins/residues, respectively. *s*^*n*^ is the standard deviation of all embedding vectors. *n* indicate the total number of proteins/residues, *n*^0^ and *n*^1^ indicate the number of ordered and disordered proteins/residues, respectively. **Fig 3C** and **3D** shown the PBC results when using template-free representation, MSA-based features, and embeddings generated from pre-trained language models. From these figures, we observed that the feature representations extracted from IDP-BERT exhibit the highest correlations with disorder. These results are not surprising because there are significant differences in the distribution of amino acid biochemical properties between disordered region and ordered region in proteins (**Fig 3E** and **3F**), and the language model trained for disordered proteins captured these biochemical properties of disordered residues (**Fig 3G**).

### Language model combination and transfer learning for disorder and disorder function prediction

**Fig 4:**
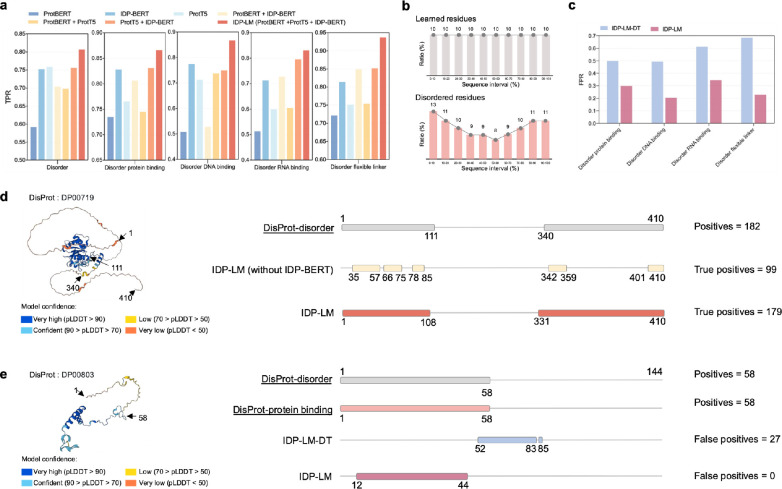
Language models combination and transfer learning improve disorder and disorder function prediction. (**a**), The true positive rates (TPR) of IDP-LMs for disorder and disorder function prediction on the validation dataset using different combinations of pre-trained protein language models. (**b**), The position distribution of the residues learned by the ProtTrans language model (upper) and the actual disordered residues in the DisPort database (lower). (**c**), The false positive rates (FPR) comparison of IDP-LMs with and without (DT) model pre-training for disorder function prediction. (**d**) and (**e**) show the prediction results of IDP-LM for two proteins in the TE176 dataset: DisProt ID: DP00719 and DisProt ID: DP00803, where the structures of two proteins were obtained by AlphaFold [45,46], and each residue in the sequences was colored based on the model confidence score, pLDDT.

To investigate how the IDP-BERT contributed to the predictions of disorder and disorder functions, we evaluated the performance of models using three language model embeddings and their different combinations on the validation dataset. **Fig 4A** shows the comparison of true positive rates (TPR) at the optimal threshold points on ROC curves of different models, from which we observed that IDP-BERT achieves more TPR compared to the other two language models, ProtBERT and ProtT5. These results contribute to the IDP- BERT being trained as a Restrictive Masked Language Model (ReMLM) to focus on the disordered regions mainly located in the head and tail segments of sequence (**Fig 4B**), thereby leading to a significant increasement of true positive predictions in the disordered regions (**Fig 4D**). We further calculated the statistical differences in propensity scores predicted by the three language models, and the results shown in **S7 and S11 Tables** demonstrated the significant differences among the three language models in disorder and disorder function predictions. Therefore, IDP-LM integrated three language models to leverage their complementary predictions, resulting in the highest predictive performance (**Fig 4A**).

The disordered function predictor IDP-LM is transferred from the model trained for disordered region prediction. To demonstrate the contribution of model transferring, we compared the performance between IDP-LM directly trained with disordered functions (IDP-LM-DT) and the fine-tuned model transferred from the pre-trained disordered region prediction, and the results were shown in **Fig 4C**. From this figure, we can see that the transfer learning significantly reduced the false positive prediction rates for all four disordered functions. The predicted results of disordered protein-binding functions for protein (DisProt ID: DP00803) from the TE176 dataset are visualized in **Fig 4E**, which indicated that the IDP-LM model transferred from disordered predictor provides fewer false positive predictions, leading to more accurate results than the IDP-LM-DT directly trained with disordered functional sequences.

### Intrinsically disordered regions/proteins prediction

**Fig 5:**
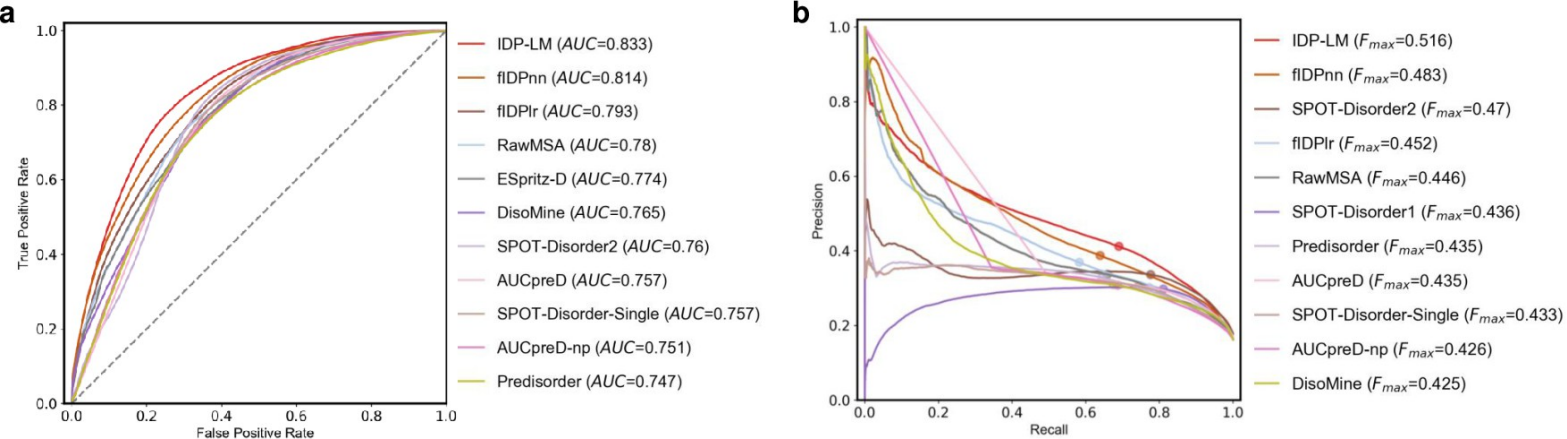
The predictive performance for IDP-LM and other disorder predictors on the CAID DisProt dataset. The ROC (**a**) and Precision–Recall (**b**) curves of IDP-LM and other ten top-ranking predictors in the CAID experiments [26]. AUC, the area under the ROC curve; F_*max*_, the maximum harmonic mean between precision and recall across all thresholds.

**Fig 6:**
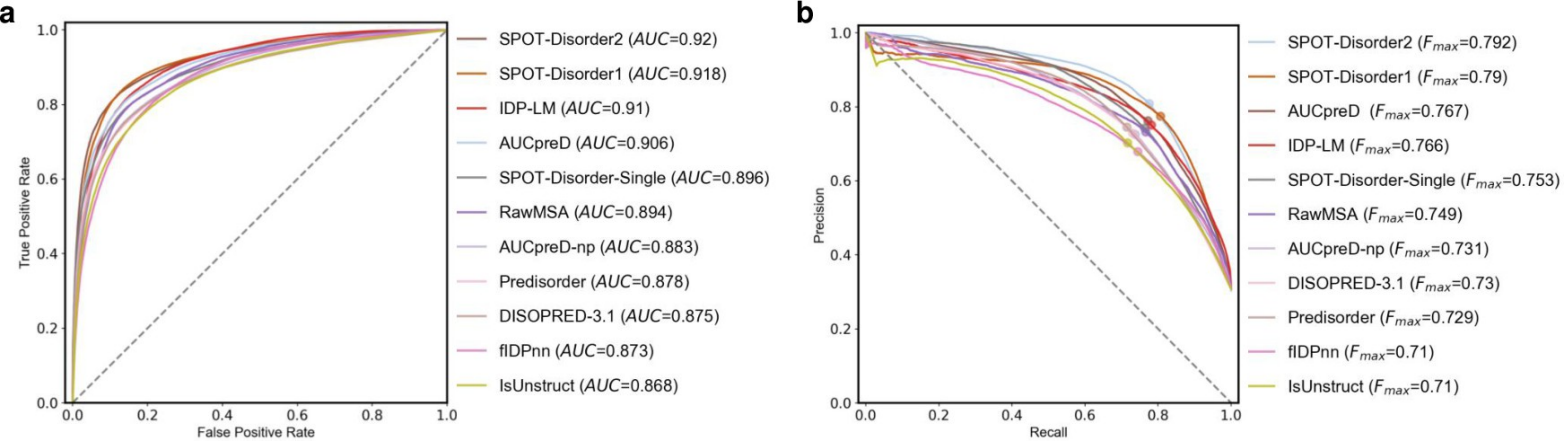
The predictive performance for IDP-LM and other disorder predictors on the CAID DisProt-PDB dataset. The ROC (**a**) and Precision–Recall (**b**) curves of IDP-LM and other ten top-ranking predictors in the CAID experiments [26]. AUC, the area under the ROC curve; F_*max*_, the maximum harmonic mean between precision and recall across all thresholds.

Following the Critical Assessment of protein Intrinsic Disorder prediction (CAID) experiment, we comprehensively evaluated the performance of IDP-LM for predicting the disorder in proteins, and compared it with other computational predictors. In CAID [26], the disorder prediction is divided into two categories: predicting intrinsically disordered regions (IDRs) in proteins and predicting fully intrinsically disordered proteins (IDPs). Two datasets are used for IDR prediction, DisProt and DisProt-PDB, where the IDR annotations in the former were collected from the DisProt database with experimental evidence, while the latter is based on the former and limited the negatives to residues observed in the PDB database. To ease comparability, we used the same evaluation metrics as in CAID to report the predictive performance of different predictors, and the IDR predictive results on DisProt and DisProt-PDB datasets were listed in **Tables 1** and **2**, respectively. From these results, we can see that the IDP-LM using the language model embeddings as exclusive input outperformed other predictors on the DisProt dataset, and achieved comparable performance with the state-of-the-art method SPOT-Disorder2 on the DisProt-PDB dataset in term of BAC values. The receiver operating characteristic (ROC) and Precision-Recall curves shown in **Figs 5** and **6** demonstrate the corresponding predictive performances. As in the CAID, proteins with at least 95% of disordered residues are considered IDPs. The performance comparisons of different predictors for identifying IDPs in the DisProt dataset were listed in **Table 3**, from which we see that the IDP-LM predictor significantly outperformed. These results of IDP-LM are attributed to the fact that the pre-trained language model learned structure information from an enormous quantity of sequences, and by combining the disordered language model captured fine-grained differences between structural order and disorder, resulting in the accurate prediction of protein disorder.

**Table 1:** Evaluation results of IDP-LM and the ten top-ranking predictors in CAID for disordered region prediction on the CAID DisProt dataset.

Methods	AUC	F_max_	MCC	BAC
IDP-LM	0.833	0.516	0.415	0.762
fIDPnn*	0.814	0.483	0.370	0.720
fIDPlr*	0.793	0.452	0.330	0.693
RawMSA*	0.780	0.445	0.328	0.714
ESpritz-D*	0.774	0.428	0.307	0.703
DisoMine*	0.765	0.424	0.299	0.698
SPOT-Disorder2*	0.760	0.469	0.349	0.725
AUCpreD*	0.757	0.433	0.318	0.712
SPOT-Disorder-Single*	0.757	0.432	0.315	0.710
AUCpreD-np*	0.751	0.424	0.301	0.699
Predisorder*	0.747	0.435	0.301	0.691

* The results of corresponding predictors were obtained from [26] evaluated on the same CAID DisProt dataset. Predictors are sorted by their AUC values.

**Table 2:** Evaluation results of IDP-LM and the ten top-ranking predictors in CAID for disordered region prediction on the CAID DisProt-PDB dataset.

Methods	AUC	F_max_	MCC	BAC
SPOT-Disorder2*	0.920	0.792	0.706	0.836
SPOT-Disorder1*	0.918	0.790	0.696	0.846
IDP-LM	0.910	0.766	0.662	0.836
AUCpreD*	0.906	0.767	0.662	0.816
SPOT-Disorder-Single*	0.896	0.753	0.646	0.817
RawMSA*	0.894	0.749	0.635	0.815
AUCpreD-np*	0.883	0.731	0.615	0.797
Predisorder*	0.878	0.729	0.619	0.788
DISOPRED-3.1*	0.875	0.730	0.613	0.796
fIDPnn*	0.873	0.710	0.576	0.782
IsUnstruct*	0.868	0.710	0.585	0.779

* The results of corresponding predictors were obtained from [26] evaluated on the same CAID DisProt-PDB dataset. Predictors are sorted by their AUC values.

**Table 3:** Evaluation results of IDP-LM and the ten top-ranking predictors in CAID for predicting fully disordered proteins on the CAID DisProt dataset.

Methods	F_max_	TN	FP	FN	TP	MCC	TNR	TPR	PPV	BAC
IDP-LM	0.680	588	19	12	33	0.657	0.969	0.733	0.635	0.851
fIDPnn*	0.598	585	16	19	26	0.569	0.973	0.578	0.619	0.776
RawMSA*	0.578	582	19	19	26	0.546	0.968	0.578	0.578	0.773
VSL2B*	0.505	578	23	22	23	0.468	0.962	0.511	0.500	0.736
fIDPlr*	0.505	566	35	18	27	0.468	0.942	0.600	0.435	0.771
Predisorder*	0.500	589	12	26	19	0.479	0.980	0.422	0.613	0.701
SPOT-Disorder1*	0.458	572	29	23	22	0.416	0.952	0.489	0.431	0.720
DisoMine*	0.455	551	50	17	28	0.421	0.917	0.622	0.359	0.770
AUCpreD*	0.453	588	13	28	17	0.431	0.978	0.378	0.567	0.678
SPOT-Disorder2*	0.452	574	27	24	21	0.409	0.955	0.467	0.438	0.711
SPOT-Disorder-Single*	0.448	594	7	30	15	0.452	0.988	0.333	0.682	0.661

* The results of corresponding predictors were obtained from [26] evaluated on the same CAID DisProt dataset. Predictors are sorted by their AUC values. TNR, true negative rate; TPR, true positive rate; PPV, positive predictive value, *i*.*e*., precision.

We obtained the confidence scores (pLDDT) produced by AlphaFold for all sequences in the CAID test dataset from the AlphaFold DB [45,46] and calculated the pLDDT distributions of the true and predicted disordered regions in the dataset (see **S1 Fig**). From this figure, we can observer that the majority of predicted disordered regions exhibit low or very low pLDDT scores, which is consistent with the true disordered regions. And the Pearson correlation coefficient between the disorder propensity scores predicted by IDP-LM and the pLDDT of AlphaFold2 was r = -0.307 (see **S5 Table**).

**Fig 7:**
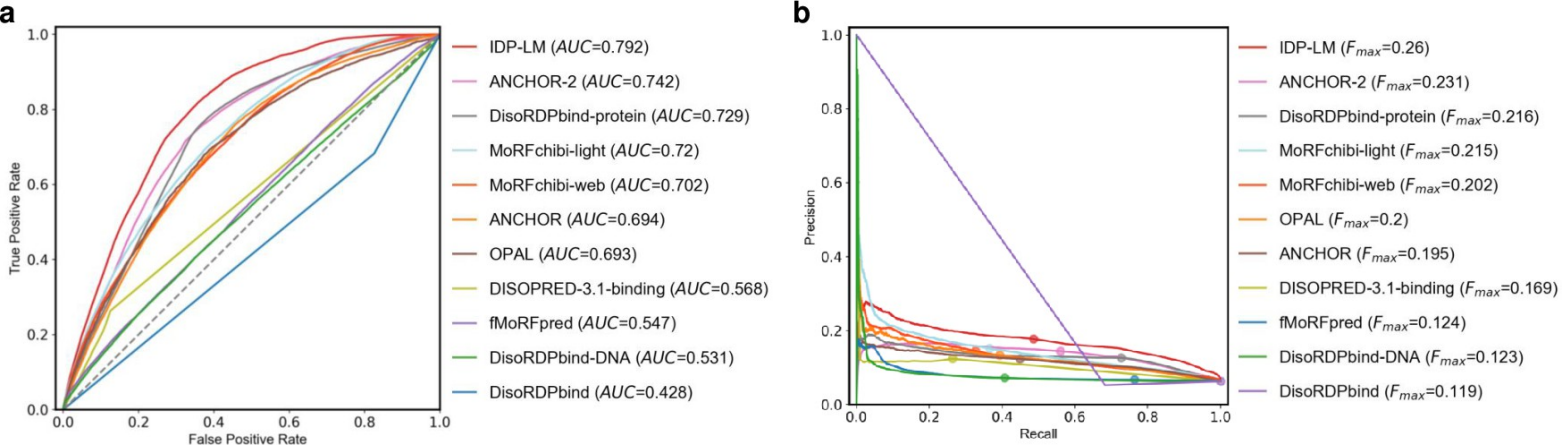
The predictive performance for IDP-LM and other binding predictors on the CAID DisProt-binding dataset. The ROC (**a**) and Precision–recall (**b**) curves of IDP-LM and other ten top-ranking predictors in the CAID experiments [26]. AUC, the area under the ROC curve; F_*max*_, the maximum harmonic mean between precision and recall across all thresholds.

Another major challenge of CAID is to predict the binding sites in protein disordered regions. The disordered binding sites are short interacting subregions in proteins, which are annotated as the features of disorder [47]. We evaluated the performance of IDP-LM predictor on the DisProt-binding dataset from CAID. The comparison results of IDP-LM and other methods are shown in **Fig 7** and **Table 4**, from which we can see that the IDP-LM predictor achieved significantly outstanding results than other predictors in all evaluation metrics, demonstrating the application of pre-trained protein language models is useful for the special target regions prediction found within IDRs.

**Table 4:** Evaluation results of IDP-LM and the ten top-ranking predictors in CAID for disordered binding sites prediction on the CAID DisProt-binding dataset.

Methods	AUC	F_max_	MCC	BAC
IDP-LM	0.792	0.260	0.239	0.730
ANCHOR-2*	0.742	0.231	0.199	0.694
DisoRDPbind-protein*	0.729	0.216	0.198	0.697
MoRFchibi-light*	0.720	0.215	0.161	0.636
MoRFchibi-web*	0.702	0.202	0.143	0.631
ANCHOR*	0.694	0.200	0.148	0.651
OPAL*	0.693	0.195	0.151	0.652
DISOPRED-3.1-binding*	0.568	0.169	0.099	0.569
fMoRFpred*	0.547	0.124	0.054	0.515
DisoRDPbind-DNA*	0.531	0.123	0.052	0.502
DisoRDPbind*	0.428	0.119	0.000	0.500

* The results of corresponding predictors were obtained from [26] evaluated on the same CAID DisProt-binding dataset. Predictors are sorted by their AUC values.

### Disordered function prediction

We compared the proposed IDP-LM predictor with the recent state-of-the-art methods for predicting four common disorder functions. The evaluation results of four functions on disorder protein binding, DNA binding, RNA binding, and flexible linker, by different predictors were listed in **Tables 5–8**. From these tables, we can see that the IDP-LM and fIDPnn predictors [27] provided all four common functional predictions for IDRs, where the fIDPnn predictor aggregated various structural and functional features at residue, window and protein levels, and achieved the second-best performance. IDP-LM, utilizing the pre-trained language model embeddings as exclusive inputs, performed best among comparable methods for all four common disordered functions. These results of IDP-LM are not surprising, because the disorder and disordered functional properties of protein maintained in their amino acid sequences, the protein language models pre-trained with massive disordered protein sequences learning these key structural and functional features. The IDP-LM takes advantage of the protein language models and maps the disorder-to-function by transfer learning from the disordered predictor, leading to the accurate predictions of four common disorder functions.

**Table 5:** Performance of IDP-LM and other predictors for disordered protein binding function prediction on the TE167 dataset.

Methods	AUC	F_max_	MCC
IDP-LM	0.824	0.473	0.403
flDPnn*	0.792	0.436	0.363
DisoRDPbind*	0.759	0.177	0.084
ANCHOR-2*	0.705	0.328	0.220
MorfChibiLight*	0.680	0.269	0.160
fMoRFpred*	0.535	0.066	0.036
MorfChibi*	0.521	0.203	0.009

* The results of corresponding predictors were obtained from [27] evaluated on the same TE167 dataset. Predictors are sorted by their AUC values.

**Table 6:** Performance of IDP-LM and other predictors for disordered DNA binding function prediction on the TE167 dataset.

Methods	AUC	F_max_	MCC
IDP-LM	0.897	0.176	0.208
flDPnn*	0.872	0.151	0.211
DisoRDPbind*	0.676	0.085	0.086

* The results of corresponding predictors were obtained from [27] evaluated on the same TE167 dataset. Predictors are sorted by their AUC values.

**Table 7:** Performance of IDP-LM and other predictors for disordered RNA binding function prediction on the TE167 dataset.

Methods	AUC	F_max_	MCC
IDP-LM	0.883	0.262	0.259
flDPnn*	0.861	0.178	0.195
DisoRDPbind*	0.647	0.133	0.126

* The results of corresponding predictors were obtained from [27] evaluated on the same TE167 dataset. Predictors are sorted by their AUC values.

**Table 8:** Performance of IDP-LM and other predictors for disordered flexible linker prediction on the TE167 dataset.

Methods	AUC	F_max_	MCC
IDP-LM	0.748	0.263	0.250
flDPnn*	0.712	0.183	0.168
DFLpred*	0.443	0.000	-0.003

* The results of corresponding predictors were obtained from [27] evaluated on the same TE167 dataset. Predictors are sorted by their AUC values.

## Conclusion

We proposed IDP-LM, a computational predictor for protein intrinsic disorder and disorder functions. The IDP-LM takes the embeddings extracted from three pre-trained protein language models as the exclusive inputs, including ProtBERT and ProtT5 and a disordered specific language model (IDP-BERT). The IDP-BERT provides fine-grained feature representations for disorder at both the residue and sequence levels. The combination of ProtBERT and ProtT5 and the disordered language model IDP-BERT provides comprehensive representations for disordered protein, which facilitates IDP-LM outperforming other comparable methods for intrinsic disorder prediction in the CAID experiments. We transferred the trained IDP-LM disorder predictor into four disorder functional predictors, including disorder protein binding, DNA binding, RNA binding, and disorder flexible linkers. Benefiting from model transfer, the IDP-LM made fewer false positives and provided high-quality prediction results for all four common disorder functions. We released the source codes for IDP-LM at https://github.com/YihePang/IDP-LM, and we also provided a stand-alone package of IDP-LM at http://bliulab.net/IDP_LM/.

## Supporting information

S1 Fig The distribution of AlphaFold confidence scores (pLDDT) in the disordered regions of CAID dataset. The real-labelled and predicted disordered regions by IDP-LM are shown in (a) and (b), respectively. The predicted disordered regions were obtained by setting the threshold for propensity scores to 0.352, which is the optimal value with maximum F1 value.
(TIF)

S1 Table The description of the disorder function benchmark datasets. (DOCX)

S2 Table The hyper-parameters of IDP-BERT. (DOCX)

S3 Table The hyper-parameters of IDP-LM for disorder prediction. (DOCX)

S4 Table The hyper-parameters of IDP-LM for disorder function prediction. (DOCX)

S5 Table Pearson correlation analysis between disorder propensity scores predicted by IDP-LM and per-residue confidence score (pLDDT) produced by AlphaFold on the CAID dataset. (DOCX)

S6 Table The differences in annotations of four disordered functions on the TE176 dataset measured by Pearson Chi-Square (Χ^2^) test. (DOCX)

S7 Table The statistical difference (P-value) between IDP-LM, ProtBERT, ProtT5, and IDP-BERT in predicting disorder on the validation dataset. (DOCX)

S8 Table The statistical difference (P-value) between IDP-LM, ProtBERT, ProtT5, and IDP-BERT in predicting disordered protein-binding on the validation dataset. (DOCX)

S9 Table The statistical difference (P-value) between IDP-LM, ProtBERT, ProtT5, and IDP-BERT in predicting disordered DNA-binding on the validation dataset. (DOCX)

S10 Table The statistical difference (P-value) between IDP-LM, ProtBERT, ProtT5, and IDP-BERT in predicting disordered RNA-binding on the validation dataset. (DOCX)

S11 Table The statistical difference (P-value) between IDP-LM, ProtBERT, ProtT5, and IDP-BERT in predicting disordered flexible linker on the validation dataset. (DOCX)

S1 Data The numerical data used in all figures. (XLSX)
