# Supplementary figures and images for: IDP-LM: Prediction of protein intrinsic disorder and disorder functions based on language models
Source: PLoS Comput Biol. 2023 Nov 22;19(11):e1011657. doi: 10.1371/journal.pcbi.1011657 (PMC10699601; doi:10.1371/journal.pcbi.1011657)

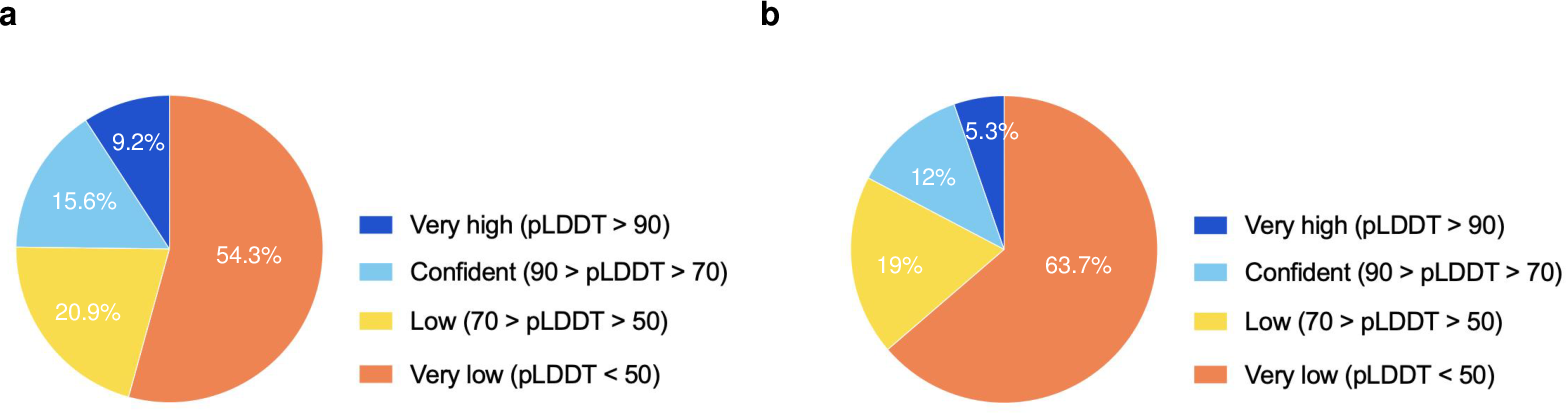

Supplement: S1 Fig — The real-labelled and predicted disordered regions by IDP-LM are shown in (a) and (b), respectively. The predicted disordered regions were obtained by setting the threshold for propensity scores to 0.352, which is the optimal value with maximum F1 value. (TIF) [file pcbi.1011657.s001.tif]
