# Supplementary material for: IDP-LM: Prediction of protein intrinsic disorder and disorder functions based on language models
Source: PLoS Comput Biol. 2023 Nov 22;19(11):e1011657. doi: 10.1371/journal.pcbi.1011657 (PMC10699601; doi:10.1371/journal.pcbi.1011657)
Supplement: S1 Table — (DOCX) [file pcbi.1011657.s002.docx]

**Table S1.** The description of the disorder function benchmark datasets.

|  | | **Dataset** | | |
| --- | --- | --- | --- | --- |
|  |  | **Train** | **Valid** | **TE176** |
| Number of residues | ALL | 202118 | 26844 | 66546 |
|  | Disorder | 45655 | 8539 | 17871 |
|  | Protein binding | 21545 | 3770 | 7855 |
|  | DNA binding | 2801 | 195 | 464 |
|  | RNA binding | 2801 | 195 | 464 |
|  | Flexible linker | 2778 | 317 | 1337 |
| Number of sequences | ALL | 412 | 90 | 176 |
|  | Disorder | 412 | 90 | 176 |
|  | Protein binding | 163 | 36 | 63 |
|  | DNA binding | 17 | 4 | 6 |
|  | RNA binding | 17 | 4 | 6 |
|  | Flexible linker | 57 | 8 | 23 |
