# Supplementary material for: IDP-LM: Prediction of protein intrinsic disorder and disorder functions based on language models
Source: PLoS Comput Biol. 2023 Nov 22;19(11):e1011657. doi: 10.1371/journal.pcbi.1011657 (PMC10699601; doi:10.1371/journal.pcbi.1011657)
Supplement: S2 Table — (DOCX) [file pcbi.1011657.s003.docx]

**Table S2.** The hyper-parameters of IDP-BERT.

| **Hyper-parameter** | |
| --- | --- |
| BERT | attention_probs_dropout_prob: 0.1 |
|  | hidden_act: ‘gelu’ |
|  | hidden_dropout_prob: 0.1 |
|  | hidden_size: 1024 |
|  | initializer_range: 0.02 |
|  | intermediate_size: 1024 |
|  | max_position_embeddings: 512 |
|  | num_attention_heads: 8 |
|  | num_hidden_layers: 6 |
|  | type_vocab_size: 2 |
|  | vocab_size: 29 |
| Mask language model | masked_lm_prob: 0.05 |
|  | max_predictions_per_seq: 50 |
|  | sampling_interval: 0.2 |
| Training settings | num_train_epochs: 50 |
|  | batch_size: 8 |
|  | warmup_proportion: 0.1 |
|  | learning_rate: 0.0001 |
