# Supplementary material for: IDP-LM: Prediction of protein intrinsic disorder and disorder functions based on language models
Source: PLoS Comput Biol. 2023 Nov 22;19(11):e1011657. doi: 10.1371/journal.pcbi.1011657 (PMC10699601; doi:10.1371/journal.pcbi.1011657)
Supplement: S3 Table — (DOCX) [file pcbi.1011657.s004.docx]

**Table S3.** The hyper-parameters of IDP-LM for disorder prediction.

| **Hyper-parameter** | |
| --- | --- |
| Prediction layers for ProtBERT | hidden_size: 128 |
|  | hidden_size for output layer: 64 |
|  | dropout_prob: 0.3 |
| Prediction layers for ProtT5 | hidden_size: 128 |
|  | hidden_size for output layer: 64 |
|  | dropout_prob: 0.3 |
| Prediction layers for IDP-BERT | hidden_size: 128 |
|  | hidden_size for output layer: 64 |
|  | dropout_prob: 0.3 |
| Training settings | num_train_epochs: 50 |
|  | batch_size: 32 |
|  | learning_rate: 0.001 |
