# Supplementary material for: IDP-LM: Prediction of protein intrinsic disorder and disorder functions based on language models
Source: PLoS Comput Biol. 2023 Nov 22;19(11):e1011657. doi: 10.1371/journal.pcbi.1011657 (PMC10699601; doi:10.1371/journal.pcbi.1011657)
Supplement: S5 Table — (DOCX) [file pcbi.1011657.s006.docx]

**Table S5.** Pearson correlation analysis between disorder propensity scores predicted by IDP-LM and per-residue confidence score (pLDDT) produced by AlphaFold on the CAID dataset.

|  | **pLDDT*** | **Propensity score*** | **r** | ***P*** |
| --- | --- | --- | --- | --- |
| Disordered region | 53.529±21.459 | 0.412±0.181 | -0.307 | <0.001 |

^*^ mean±standard deviation
