# Supplementary material for: IDP-LM: Prediction of protein intrinsic disorder and disorder functions based on language models
Source: PLoS Comput Biol. 2023 Nov 22;19(11):e1011657. doi: 10.1371/journal.pcbi.1011657 (PMC10699601; doi:10.1371/journal.pcbi.1011657)
Supplement: S6 Table — (DOCX) [file pcbi.1011657.s007.docx]

**Table S6.** The differences in annotations of four disordered functions on the TE176 dataset measured by Pearson Chi-Square (Χ²) test.

|  | **Protein binding** | | **DNA binding** | | **RNA binding** | | **Flexible linker** | |
| --- | --- | --- | --- | --- | --- | --- | --- | --- |
|  | $\chi^{2}$ | *P* | $\chi^{2}$ | *P* | $\chi^{2}$ | *P* | $\chi^{2}$ | *P* |
| **Protein binding** | / | / | 144.413 | <0.001 | 144.413 | <0.001 | 74.522 | <0.001 |
| **DNA binding** | 144.413 | <0.001 | / | / | 66546.0 | <0.001 | 9.580 | 0.003 |
| **RNA binding** | 144.413 | <0.001 | 66546.0 | <0.001 | / | / | 9.580 | 0.003 |
| **Flexible linker** | 74.522 | <0.001 | 9.580 | 0.003 | 9.580 | 0.003 | / | / |

^*^ A higher $\chi^{2}$ and *P*<0.005 indicate more significant differences.
