# Supplementary material for: IDP-LM: Prediction of protein intrinsic disorder and disorder functions based on language models
Source: PLoS Comput Biol. 2023 Nov 22;19(11):e1011657. doi: 10.1371/journal.pcbi.1011657 (PMC10699601; doi:10.1371/journal.pcbi.1011657)
Supplement: S7 Table — (DOCX) [file pcbi.1011657.s008.docx]

**Table S7.** The statistical difference (*P*-value) between IDP-LM, ProtBERT, ProtT5, and IDP-BERT in predicting disorder on the validation dataset.

| **Disorder** | **ProtBERT** | **ProtT5** | **IDP-BERT** | **IDP-LM** |
| --- | --- | --- | --- | --- |
| **ProtBERT** | / | 2.108E-108 | 3.946E-8 | 8.296E-74 |
| **ProtT5** | 2.108E-108 | / | 8.352E-145 | 2.453E-6 |
| **IDP-BERT** | 3.946E-8 | 8.352E-145 | / | 4.217E-108 |
| **IDP-LM** | 8.296E-74 | 2.453E-6 | 4.217E-108 | / |
