# Supplementary material for: IDP-LM: Prediction of protein intrinsic disorder and disorder functions based on language models
Source: PLoS Comput Biol. 2023 Nov 22;19(11):e1011657. doi: 10.1371/journal.pcbi.1011657 (PMC10699601; doi:10.1371/journal.pcbi.1011657)
Supplement: S8 Table — (DOCX) [file pcbi.1011657.s009.docx]

**Table S8.** The statistical difference (*P*-value) between IDP-LM, ProtBERT, ProtT5, and IDP-BERT in predicting disordered protein-binding on the validation dataset.

| **Disordered protein binding** | **ProtBERT** | **ProtT5** | **IDP-BERT** | **IDP-LM** |
| --- | --- | --- | --- | --- |
| **ProtBERT** | / | 3.900E-75 | 0.000E+00 | 0.000E+00 |
| **ProtT5** | 3.900E-75 | / | 0.000E+00 | 0.000E+00 |
| **IDP-BERT** | 0.000E+00 | 0.000E+00 | / | 0.000E+00 |
| **IDP-LM** | 0.000E+00 | 0.000E+00 | 0.000E+00 | / |
