# Supplementary material for: IDP-LM: Prediction of protein intrinsic disorder and disorder functions based on language models
Source: PLoS Comput Biol. 2023 Nov 22;19(11):e1011657. doi: 10.1371/journal.pcbi.1011657 (PMC10699601; doi:10.1371/journal.pcbi.1011657)
Supplement: S9 Table — (DOCX) [file pcbi.1011657.s010.docx]

**Table S9.** The statistical difference (*P*-value) between IDP-LM, ProtBERT, ProtT5, and IDP-BERT in predicting disordered DNA-binding on the validation dataset.

| **Disordered DNA binding** | **ProtBERT** | **ProtT5** | **IDP-BERT** | **IDP-LM** |
| --- | --- | --- | --- | --- |
| **ProtBERT** | / | 2.150E-17 | 3.632E-12 | 3.587E-13 |
| **ProtT5** | 2.150E-17 | / | 2.435E-9 | 1.240E-4 |
| **IDP-BERT** | 3.632E-12 | 2.435E-9 | / | **1.130E-1**^*^ |
| **IDP-LM** | 3.587E-13 | 1.240E-4 | **1.130E-1**^*^ | / |

^*^ Bold font identifies *P*-values > 0.05.
