# Supplementary material for: IDP-LM: Prediction of protein intrinsic disorder and disorder functions based on language models
Source: PLoS Comput Biol. 2023 Nov 22;19(11):e1011657. doi: 10.1371/journal.pcbi.1011657 (PMC10699601; doi:10.1371/journal.pcbi.1011657)
Supplement: S10 Table — (DOCX) [file pcbi.1011657.s011.docx]

**Table S10.** The statistical difference (*P*-value) between IDP-LM, ProtBERT, ProtT5, and IDP-BERT in predicting disordered RNA-binding on the validation dataset.

| **Disordered RNA binding** | **ProtBERT** | **ProtT5** | **IDP-BERT** | **IDP-LM** |
| --- | --- | --- | --- | --- |
| **ProtBERT** | / | 4.034E-4 | 4.215E-4 | 3.610E-4 |
| **ProtT5** | 4.034E-4 | / | 6.862E-3 | **8.094E-1**^*^ |
| **IDP-BERT** | 4.215E-4 | 6.862E-3 | / | 7.734E-3 |
| **IDP-LM** | 3.610E-4 | **8.094E-1**^*^ | 7.734E-3 | / |

^*^ Bold font identifies *P*-values > 0.05.
