# Supplementary material for: IDP-LM: Prediction of protein intrinsic disorder and disorder functions based on language models
Source: PLoS Comput Biol. 2023 Nov 22;19(11):e1011657. doi: 10.1371/journal.pcbi.1011657 (PMC10699601; doi:10.1371/journal.pcbi.1011657)
Supplement: S11 Table — (DOCX) [file pcbi.1011657.s012.docx]

**Table S11.** The statistical difference (*P*-value) between IDP-LM, ProtBERT, ProtT5, and IDP-BERT in predicting disordered flexible linker on the validation dataset.

| **Disordered flexible linker** | **ProtBERT** | **ProtT5** | **IDP-BERT** | **IDP-LM** |
| --- | --- | --- | --- | --- |
| **ProtBERT** | / | 1.888E-67 | 3.596E-114 | 4.803E-81 |
| **ProtT5** | 1.888E-67 | / | 1.238E-6 | **5.887E-1**^*^ |
| **IDP-BERT** | 3.596E-114 | 1.238E-6 | / | 1.805E-6 |
| **IDP-LM** | 4.803E-81 | **5.887E-1**^*^ | 1.805E-6 | / |

^*^ Bold font identifies *P*-values > 0.05.
